# Prevalence of Low Back Pain and Associated Factors Among Family Caregivers of Patients With Disabilities in Shenzhen, China: A Cross‐Sectional Study Based on Binary Logistic Regression Modelling

**DOI:** 10.1002/nop2.70787

**Published:** 2026-09-22

**Authors:** Yu‐Jiao Sun, Li Dai, Jiao Xiong, Su Liang

**Affiliations:** ^1^ Department of Endocrinology The People's Hospital of Baoan Shenzhen Shenzhen Guangdong China; ^2^ Department of Home Health Care Services The People's Hospital of Baoan Shenzhen Shenzhen Guangdong China; ^3^ School of Nursing Guangxi University of Chinese Medicine Nanning Guangxi China

**Keywords:** associated factors, disabled patients, family caregiver, low back pain, safe work behaviour, social support

## Abstract

**Aim:**

To examine the prevalence of low back pain among family caregivers of patients with disabilities and identify its associated factors.

**Design:**

Cross‐sectional study.

**Methods:**

From May to August 2023, 209 family caregivers of patients with disabilities were recruited from four tertiary hospitals using convenience sampling. Data were collected using a general information questionnaire, the modified Barthel Index, the Chinese version of the Safe Work Behaviour related to Patient Handling Scale, and the Chinese version of the Perceived Social Support Scale. Descriptive statistics and binary logistic regression were used. Gender, age and regular exercise were included as adjustment variables based on prior evidence and clinical relevance.

**Results:**

LBP was reported by 117 family caregivers, corresponding to a prevalence of 55.98%. In the adjusted model, family caregivers who repositioned patients 4 to 7 times per day had higher odds of LBP than those who repositioned patients no more than 3 times per day (adjusted OR = 3.444, 95% CI = 1.279 to 9.270). Family caregivers who repositioned patients at least 8 times per day also had higher odds of LBP (adjusted OR = 4.821, 95% CI = 1.984 to 11.714). Each additional year of caregiving was associated with higher odds of LBP (adjusted OR = 1.119, 95% CI = 1.036 to 1.209). High patient handling safety behaviour was associated with lower odds of LBP (adjusted OR = 0.254, 95% CI = 0.120 to 0.536). Higher perceived social support was also associated with lower odds of LBP (adjusted OR = 0.949, 95% CI = 0.925 to 0.974). The model had a Nagelkerke *R* ^2^ of 0.472.

**Conclusion:**

LBP was common among family caregivers of patients with disabilities. Longer caregiving duration, more frequent patient repositioning, lower patient‐handling safety behaviour and lower perceived social support were associated with LBP. Healthcare professionals should consider these factors when assessing family caregivers and provide appropriate patient‐handling training and supportive resources; however, longitudinal and interventional studies are needed before causal or preventive effects can be established.

**Implications for Nursing Practice:**

Nurses should assess caregiving duration, patient repositioning frequency, patient handling safety behaviour and perceived social support during discharge planning and community follow up. Family caregivers who may require additional support should receive individualized safe handling training and referral to appropriate resources.

**Patient or Public Contribution:**

No Patient or Public Contribution.

## 1 Introduction

Low back pain (LBP) is a leading cause of disability worldwide and contributes substantially to the global burden of musculoskeletal conditions (World Health Organization 2024). According to the World Health Organization (2023), the condition for which the greatest number of people benefit from rehabilitation is lower back pain (LBP), which is the primary cause of disability globally. Chou (2010) defined low back pain as ‘pain, muscle tension, or stiffness localized above the inferior gluteal folds and below the costal margin with or without leg pain’. The quality of life and productivity at work can be greatly impacted by LBP, which can also result in chronic pain, dysfunction and even disability (Davey et al. 2019; Gomes et al. 2019).

## 2 Background

China has a large and growing population of individuals with disabilities. It is projected that by 2030, the total number of disabled individuals in China will reach 136.24–136.74 million (Luo et al. 2021). Due to their limited ability to perform daily activities, patients with disabilities require frequent assistance, which leads to care dependence. Under the influence of traditional cultural values and the limited implementation of the long‐term care insurance system, approximately 90% of disabled individuals rely on long‐term family care (Gu et al. 2018). This results in an increasing number of family caregivers and a growing burden on them (Liao 2019).

Family caregivers often assume physically demanding roles that involve prolonged bending, twisting and lifting, all of which predispose them to the development of low back pain (LBP) (Muthukrishnan and Maqbool Ahmad 2021; Cottagiri and Sykes 2019). These family caregivers are often untrained, lacking knowledge of proper lifting techniques or back safety, and have a low level of literacy regarding injury prevention (Tao and McRoy 2015). LBP is a common consequence of caregiving activities such as bending, twisting, heavy lifting, maintaining awkward postures and experiencing psychological stress (Pajeemas et al. 2018; Yilmaz Yalcinkaya et al. 2010).

Disability affects the individual with the disability as well as the whole family because people with disabilities need assistance with everyday tasks and medicine administration (Ghazawy et al. 2020). For the purposes of this study, low back pain is defined as experiencing discomfort such as pain, muscle tension or stiffness in the back below the lower edge of the ribs and above the buttock crease within the past 1 year, and this pain or discomfort has been severe enough to limit your daily activities or change your daily routine for at least 1 day, excluding fever and menstrual‐induced pain (De Vet, Heymans, Dunn, et al. 2002; Dionne et al. 2008).

Family caregivers of patients with disabilities present different prevalence rates of LBP across the literature mentioned that LBP occurs depending on the degree of dependency of the disabled patient, family caregiver characteristics, and caregiving tasks. A survey by Fagerström et al. (2020) in Sweden found that the occurrence of LBP in elderly family caregivers was 56.7%. Two studies, Nigerian and Turkish studies, showed that 28%–82.8% of family caregivers of stroke patients develop low back pain (Abba et al. 2022; Yilmaz Yalcinkaya et al. 2010).

Numerous studies have elaborated that LBP in family caregivers is related to their factors, caregiving‐related factors and social support factors. Their demographic characteristics, such as female, age, literacy, relationship with the patient, and other factors, have been described in many studies (Gomes et al. 2019; Jeon et al. 2017). It has been found that unfavourable factors in caregiving: excessive patient dependence, poor protection awareness, solitary caregiving, unfavourable ergonomic factors such as heavy lifting, poor labour posture, repetitive manipulation and other caregiving factors are correlated with the occurrence of LBP (Darragh et al. 2015; Gomes et al. 2019; Muthukrishnan and Maqbool Ahmad 2021). In addition, a study on family caregiver social support showed that family caregivers' perceived social support was positively associated with their health status (Nicolson et al. 2021). Individuals with inadequate perceived social support were at risk for limited physical function and chronic pain (Orlas et al. 2021).

In China, research on family caregivers of patients with disabilities has focused primarily on caregiving abilities, coping styles and psychological impacts. However, there is a lack of studies specifically addressing the prevalence of LBP among this population. Given China's unique socio‐cultural context and family structure, further research is necessary to better understand the factors contributing to LBP among family caregivers of patients with disabilities.

Evidence regarding LBP among family caregivers of patients with disabilities in China remains limited. Guided by the epidemiological triangle framework, this study considered caregiver characteristics as host factors, caregiving workload and patient handling safety behaviour as exposure‐related factors, and perceived social support as an environmental factor. The study aimed to estimate the prevalence of LBP and identify factors associated with its occurrence among family caregivers of patients with disabilities.

### 2.1 Purpose

To examine the prevalence of low back pain among family caregivers of patients with disabilities and identify factors associated with its occurrence.

## 3 Methods

### 3.1 Design

This study employed an analytical cross‐sectional study design and was reported in accordance with the STROBE criteria: Strengthening the Reporting of Observational Studies in Epidemiology.

### 3.2 Sample and Participants

The target population comprised family caregivers of patients with disabilities who received care at four tertiary Grade A hospitals in Shenzhen, Guangdong Province, China. Participants were recruited using convenience sampling. Convenience sampling was used because family caregivers of patients with disabilities are not registered as an independent clinical population in routine hospital or community health records. Therefore, a complete sampling frame was unavailable. Eligible family caregivers were approached during the patients' hospitalization, discharge preparation, or follow‐up period. This approach enabled the researchers to identify family caregivers who met the eligibility criteria and were actively providing long‐term daily care.

The sample size was estimated using the single population proportion formula for cross‐sectional studies: *n = Z* ^*2*^ *p(1 − p)/d* ^2^. The pre‐survey showed that the prevalence of LBP among family caregivers was 70.9%. Therefore, *p* was set at 0.709, *Z* was set at 1.96 for a two sided α of 0.05, and the allowable error was set at 10% of *p* (Sharma et al. 2020). The minimum required sample size was 158. After increasing this number by 20% to account for possible non‐response or invalid questionnaires, the target sample size was 190. A total of 209 valid questionnaires were included in the final analysis, which exceeded the minimum requirement.

Family caregivers were eligible if they cared for a patient with disabilities whose Modified Barthel Index score was 70 or lower, were aged 18 years or older, had provided continuous care for at least 6 months and at least 8 h per day, were able to complete the questionnaire independently or with assistance from a researcher, and voluntarily agreed to participate. Family caregivers were excluded if they had LBP attributable to an accidental injury, a preexisting lumbar condition such as lumbar disc herniation, back pain associated with fever, menstruation, or pregnancy, a mental disorder or serious illness that prevented participation, or if they withdrew during the survey.

### 3.3 Data Collection and Instruments

The data collection process lasted for 4 months, from May to August 2023. Before collecting the data, the investigator explained the purpose of the study and the method of filling out the questionnaire to the respondents. The respondents were asked to sign an informed consent form before filling out the questionnaire. For respondents who were unable to complete the questionnaire independently, the investigator completed the electronic questionnaire on their behalf based on their verbal responses. This face‐to‐face questionnaire survey was conducted among family caregivers of patients with disabilities who met the inclusion criteria. All questionnaires were collected after 30 min. The data were collected using the general information questionnaire, the Modified Barthel Index Rating Scale, the Chinese Version of the Patient Handling Related Safety Behaviour Scale, and the Chinese Version of the Perceived Social Support Scale.

**General Information Questionnaire:** Based on a literature review and expert consultation, we developed a general information questionnaire that included two sections: (1) demographic characteristics of family caregivers of patients with disabilities, including age, gender, education level, marital status and regular exercise; and (2) caregiving‐related characteristics, including care years, daily care hours and frequency of patient repositioning.

**The modified Barthel Index Rating Scale** (Mahoney and Barthel 1965) was used to evaluate the ability of patients with disabilities to live their daily lives. It is divided into five grades, including complete dependence (0–20 points), severe dependence (21–45 points), moderate dependence (46–70 points), mild dependence (71–99 points) and complete self‐care (100 points). The score is 100 points, and the higher score represents better self‐care ability. The Cronbach's alpha coefficient of the questionnaire was 0.809.

**Chinese Version of the Patient Handling Related Safety Behaviour Scale** (Lee et al. 2010) developed the Patient Handling Related Safety Behaviour Scale to evaluate patients' behavioural performance in the preparation and execution phases of manual handling tasks. This scale was translated into Chinese by (Sun et al. 2024). The Chinese version of the scale consisted of 13 items divided into 2 dimensions: the preparation phase and the execution phase of manual handling tasks. The preparation phase included 8 items, and the execution phase consisted of 5 items with a 6‐point Likert scale (1 = never to 6 = always). Item 4 was a negative response.

Regarding items 8 and 9, if the height of the patient bed could not be adjusted or if there was no assisted transfer device, the response of the ‘not applicable’ option could be selected at the time of the survey, and the corresponding items were not scored. The variable was skewed in this study and was therefore dichotomized at the median, with scores at or above the median defined as ‘high security’. To examine whether dichotomization affected the findings, a sensitivity analysis was conducted by entering the SWB PH mean score as a continuous variable in the logistic regression model.

The higher the SWB‐PH (Safe Work Behaviour related to Patient Handling, SWB‐PH) score, the safer the work behaviour. At present, most of the assessment tools for evaluating human mechanical behaviour are used to investigate systemic musculoskeletal injuries in occupational groups such as workers, and there is a lack of targeting of low back pain injuries in the non‐occupational groups, and there is a lack of tools to measure caring and safe behaviours. The Chinese version of the SWB‐PH questionnaire has good reliability and validity, and the questions in the questionnaire are clear and easy to understand, which is feasible and culturally applicable, and therefore, it was chosen as the measurement tool. The content validity of the Chinese version was 0.80 to 1.00 for the I‐CVI and 0.96 for the S‐CVI. The Cronbach's alpha coefficient of the questionnaire was 0.799.

**The Chinese version of the Perceived Social Support Scale** (Jiang 2001) was employed to measure the individual's perception of support from family and friends, among others. The scale consisted of 12 items with a 7‐point Likert scale (1 = strongly disagree to 7 = strongly agree). The possible total score ranges from 12 to 84. The total score between 12 and 36 is considered low support; the total score between 37 and 60 is considered intermediate support; and the total score between 61 and 84 is considered high support. A higher total score indicates a better level of social support. The Scale has good reliability and validity and is widely used in China. In this study, the Cronbach's alpha coefficient was 0.947.

### 3.4 Statistical Analysis

Data were analysed using IBM SPSS Statistics version 24. The data were checked for missing values before analysis, and no missing data were identified. Categorical variables are presented as frequencies and percentages, whereas non‐normally distributed continuous variables are presented as medians and interquartile ranges. Group differences were examined using chi‐square tests or Wilcoxon rank‐sum tests, as appropriate. Binary logistic regression was used to examine factors associated with low back pain (LBP). Variables associated with LBP in the univariable analyses were considered for inclusion in the multivariable model. Caregiver age, gender and regular exercise were included as prespecified adjustment variables based on clinical relevance. Repositioning frequency was entered as a categorical variable, with no more than three times per day as the reference category. Care years and perceived social support were entered as continuous variables. Patient‐handling safety behaviour was dichotomized at the median, with low safety behaviour as the reference category. Model calibration was evaluated using the Hosmer–Lemeshow test, and model discrimination was assessed using the area under the receiver operating characteristic curve (AUC). Multicollinearity was assessed using tolerance and variance inflation factor (VIF) values. Tolerance values greater than 0.20 and VIF values below 5 were considered acceptable. To assess the robustness of the median split, we performed a sensitivity analysis using the continuous SWB‐PH mean score in place of the dichotomized patient handling safety behaviour variable. The sensitivity model included the same covariates as the primary model. Full results are presented in Table S1.

### 3.5 Ethical Considerations

This study was approved by the Ethics Committee of the hospital where it was conducted (BYL20220508); before the start of the study, the purpose of the study, the process of the study, the significance of the study were explained to the research subjects, the voluntary nature of participation, confidentiality and harmlessness were explained, and informed consent was signed after consent was obtained; and participants could quit the study at any time, and it would not cause the respondents any adverse effects or distress, and will not jeopardize the needs and interests of the respondents; the researcher will keep the personal data of the research subjects confidential after the completion of the survey.

## 4 Results

### 4.1 General Information on the Study Population

A total of 220 questionnaires were distributed, and 209 valid questionnaires were received with a valid respondent rate of 95.00%. The median age of the 209 participants was 52 (48, 55) years. Among 209 family caregivers, 117 reported LBP during the previous 12 months, corresponding to a prevalence of 55.98%. Details of other variables are shown in Table 1.

**TABLE 1:** General demographic information on family caregivers of patients with disabilities (*n* = 209).

Variables	No LBP group *n* = 92, *n* (%)	LBP group, *n* = 117, *n* (%)
Gender		
Male	22 (23.91)	32 (27.35)
Female	70 (76.09)	85 (72.65)
Degree of education		
Primary school and below	31 (33.70)	51 (43.59)
Junior middle school	37 (40.22)	47 (40.17)
High school and above	24 (26.09)	19 (16.24)
Marital status		
Married	92 (100.00)	115 (98.29)
Unmarried	0	2 (1.71)
Monthly household income, CNY		
< 4000	12 (13.04)	12 (10.26)
4000~	41 (44.57)	76 (64.96)
8000~	39 (42.39)	29 (24.79)
Regular exercise		
No	78 (84.78)	115 (98.29)
< 3 times per week (short exercise)	13 (14.13)	1 (0.85)
< 3 times per week (prolonged exercise)	0	0
3–5 times per week (short exercise)	1 (1.08)	0
3–5 times per week (prolonged exercise)	0	1 (0.85)

*Note:* Values are presented as *n* (%).

Abbreviation: LBP = low back pain.

### 4.2 Association Between Caregiving Factors and Occurrence of Low Back Pain

The results of the univariate analysis showed that the seven factors were associated with the occurrence of low back pain among family caregivers, as shown in Table 2, including family caregiver's age (*z* = −2.083, *p* = 0.037), number of repositioning (*χ* ^*2*^ = 35.274, *p* < 0.001), regular exercise (*χ* ^*2*^ = 13.293, *p* < 0.001), daily care hours (z = −2.068, *p* = 0.039), care years (*z* = −3.706, *p* < 0.001), safety work behaviours (*χ* ^*2*^ = 29.954, *p* < 0.001) and perceived social support (*z* = −6.595, *p* < 0.001).

**TABLE 2:** One‐way analysis of the characteristics of patients with disabilities and family caregivers (*n* = 209).

Variables	No LBP group, *n* = 92, *n* (%) or median (IQR)	LBP group, *n* = 117, *n* (%) or median (IQR)	*χ* ^*2*^ or Ζ	*p*
ADL for disabled patient (Score)			5.212^a^	0.074
Moderate dependence	31 (33.70)	32 (27.35)		
Heavy dependence	32 (34.78)	30 (25.64)		
Absolute dependence	29 (31.52)	55 (47.01)		
The number of repositioning (times/day)			35.274^a^	**< 0.001**
1–3	47 (51.09)	17 (14.53)		
4–7	21 (22.83)	31 (26.50)		
≥ 8	24 (26.09)	69 (58.97)		
Regular exercise			13.293^a^	**< 0.001**
No	78 (84.78)	115 (98.29)		
Yes	14 (15.22)	2 (1.71)		
Safe Work Behaviour			29.954^a^	**< 0.001**
Low safety	21 (22.83)	71 (60.68)		
High safety	71 (77.17)	46 (39.32)		
Age (years)	51 (48,54)	53 (48,56)	−2.083^b^	**0.037**
Care years (years)	2 (1,4)	3 (2,7)	−3.706^b^	**< 0.001**
Daily care hours (hours)	18 (17,19)	19 (18,19)	−2.068^b^	0.039
Perceived social support (score)	52 (41,63)	34 (27.5,43.5)	−6.595^b^	**< 0.001**

*Note:* Values are presented as *n* (%) for categorical variables and median (IQR) for continuous variables; *χ* ^*2*^ = Chi‐square, Z = Wilcoxon rank‐sum test; a indicates *χ* ^*2*^  value, b indicates Z value; the chi‐square test was used for categorical variables, and the Wilcoxon rank‐sum test was used for continuous variables with non‐normal distributions; Bold *p* values indicate *p* < 0.05.

Abbreviations: ADL, activities of daily living; IQR, interquartile range; LBP, low back pain.

### 4.3 Factors Associated With Low Back Pain Among Family Caregivers

**FIGURE 1:**
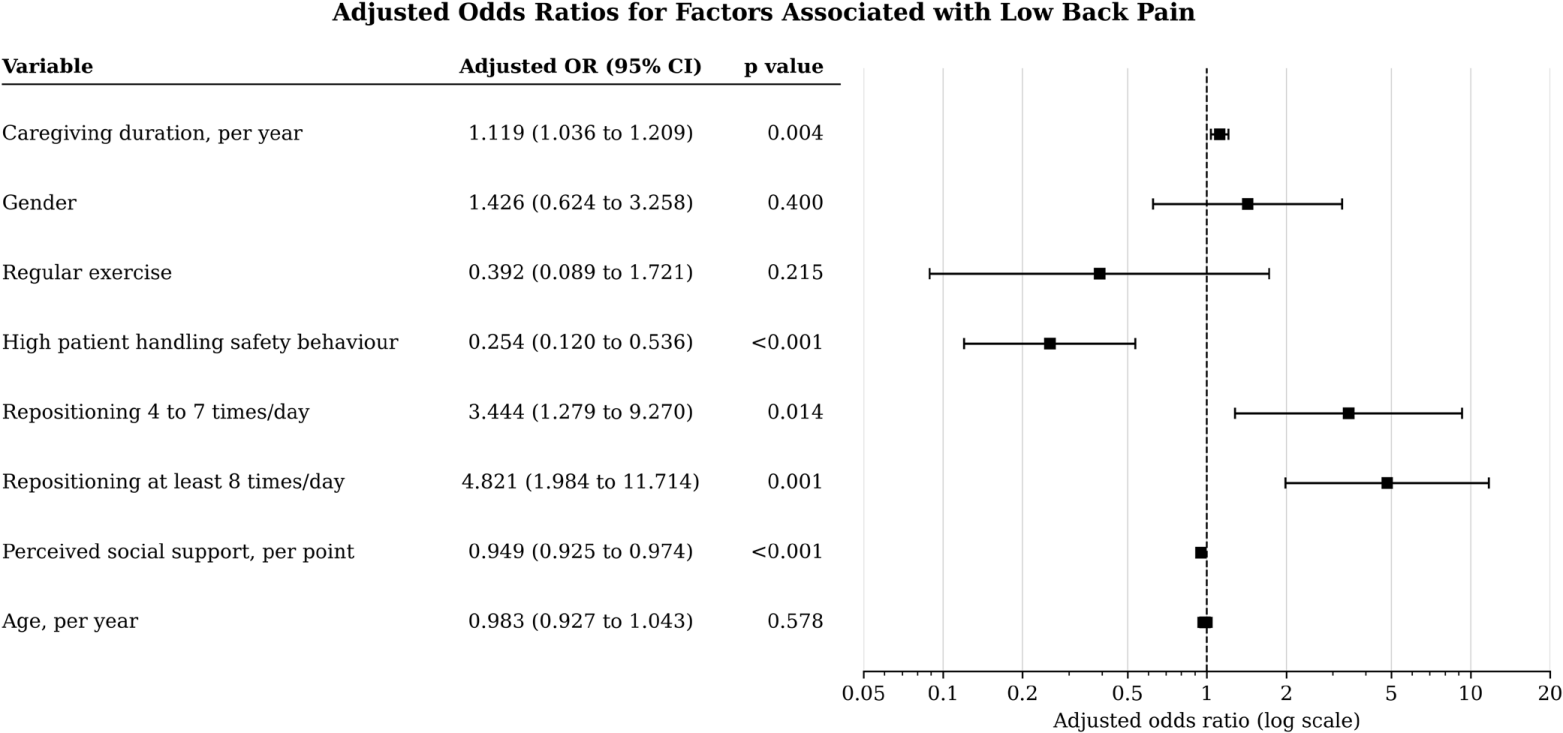
Forest plot of adjusted odds ratios for factors associated with LBP among family caregivers of patients with disabilities. Squares represent adjusted ORs, and horizontal lines represent 95% CIs. The vertical reference line indicates OR = 1. Estimates with 95% CIs that do not cross 1 were considered statistically significant when *p* < 0.05. LBP = low back pain. OR = odds ratio. CI = confidence interval.

Variable coding is presented in Table 3, and the adjusted logistic regression results are presented in Table 4 and visually summarized in Figure 1. In the adjusted model, care years, number of repositioning, patient handling safety behaviour and perceived social support were significantly associated with LBP. Compared with family caregivers who repositioned patients no more than 3 times per day, those who repositioned patients 4 to 7 times per day had higher odds of LBP (adjusted OR = 3.444, 95% CI = 1.279 to 9.270), as did those who repositioned patients at least 8 times per day (adjusted OR = 4.821, 95% CI = 1.984 to 11.714). Each additional year of caregiving was associated with higher odds of LBP (adjusted OR = 1.119, 95% CI = 1.036 to 1.209). High patient handling safety behaviour was associated with lower odds of LBP (adjusted OR = 0.254, 95% CI = 0.120 to 0.536) and higher perceived social support was associated with lower odds of LBP (adjusted OR = 0.949, 95% CI = 0.925 to 0.974). Gender, age, and regular exercise were not significantly associated with LBP. The model had a Nagelkerke *R* ^*2*^ of 0.472 and acceptable calibration (Hosmer Lemeshow *χ* ^*2*^ = 9.795, *p* = 0.280). The model showed good discrimination (AUC = 0.875, 95% CI = 0.828 to 0.923, *p* < 0.001). Tolerance values ranged from 0.777 to 0.957 and VIF values from 1.045 to 1.286, indicating no serious multicollinearity. In the sensitivity analysis, the continuous SWB‐PH mean score remained associated with lower odds of LBP (adjusted OR = 0.225, 95% CI = 0.108 to 0.469, *p* < 0.001). The sensitivity model had a Nagelkerke *R* ^*2*^ of 0.493 and acceptable calibration (Hosmer Lemeshow *χ* ^*2*^ = 10.068, *p* = 0.260). Full results are presented in Supplementary Table S1.

**TABLE 3:** Variable coding for the binary logistic regression model (*n* = 209).

Independent variable	Assignment method
Gender	Male = 0; Female = 1
Regular exercise	No = 0; Yes = 1
Safe work behaviour	< Median score = 0; ≥ Median score = 1
The number of repositioning	≤ 3Times/day = 0; 4–7Times/day = 1; ≥ 8Times/day = 2

**TABLE 4:** Adjusted logistic regression analysis of factors associated with low back pain among family caregivers of patients with disabilities (*n* = 209).

Variables	B	SE	Wald *χ* ^*2*^	*p*	Adjusted OR	95% CI
Age	−0.017	0.030	0.309	0.578	0.983	[0.927, 1.043]
Care years	0.113	0.039	8.162	0.004	1.119	[1.036, 1.209]
Gender	0.355	0.422	0.709	0.400	1.426	[0.624, 3.258]
Regular exercise	−0.938	0.755	1.540	0.215	0.392	[0.089, 1.721]
Safe work behaviour	−1.372	0.381	12.945	< 0.001	0.254	[0.120, 0.536]
The number of repositioning			12.346	0.002		
No more than 3 times per day	Ref	Ref	Ref	Ref	Ref	Ref
4 to 7 times per day	1.237	0.505	5.991	0.014	3.444	[1.279, 9.270]
At least 8 times per day	1.573	0.453	12.058	0.001	4.821	[1.984, 11.714]
Perceived social support	−0.052	0.013	16.120	< 0.001	0.949	[0.925, 0.974]

*Note:* Model fit: Nagelkerke *R* ^2^ = 0.472 and Hosmer Lemeshow *χ* ^*2*^ = 9.795, *p* = 0.280. AUC = 0.875, 95% CI = 0.828 to 0.923, *p* < 0.001. Tolerance values ranged from 0.777 to 0.957 and VIF values ranged from 1.045 to 1.286.

Abbreviations: Adjusted OR, adjusted odds ratio; CI, confidence interval; LBP, low back pain; Ref, reference category; SE, standard error.

## 5 Discussion

Interpreted through the epidemiological triangle framework, the findings suggest that LBP among family caregivers is shaped by the combined effects of cumulative caregiving exposure, unsafe patient‐handling behaviours and insufficient social support. This study found that the prevalence of low back pain among family caregivers of patients with disabilities was 55.98% (*n* = 117). It was lower than the study done in Thailand, in which 64.6% of family caregivers of dependent spinal cord lesion patients had low back pain (Pajeemas et al. 2018). However, the finding was inconsistent with a study in Japan by Hasuo et al. (2020), which may have contributed to the variation in low back pain prevalence in that it discovered 28.3% of chronic low back pain among cancer patients' family caregivers. Another study in Japan found that the prevalence of low back pain among family caregivers was 69% (Abiko et al. 2024). These variations highlight potential differences in the prevalence of low back pain among family caregivers across different samples and regions.

Repositioning frequency was strongly associated with LBP among family caregivers. Compared with family caregivers who repositioned patients ≤ 3 times per day, those who repositioned patients 4 to 7 times per day and ≥ 8 times per day had higher odds of LBP. This finding is consistent with previous studies showing that caregiving tasks involving body repositioning, bending, twisting, lifting and repeated lumbar flexion are related to LBP among family caregivers (Suzuki et al. 2016; Ramezani et al. 2020). Frequent repositioning may increase cumulative lumbar loading and muscle fatigue, particularly when family caregivers perform these tasks without adequate technique, assistance, or equipment.

The second most significant factor was caring years. The results of this study showed that the odds of LBP in family caregivers would increase by 1.119 times for every 1‐year increase in years of care. This is consistent with a previous study done in Nigeria by Abdullahi et al. (2022), which found that caregiving for 5 h or more per day significantly predicted the presence of low back pain among family caregivers of stroke survivors. These results are also consistent with an observational study by Vega‐Vélez et al. (2021), which included 283 family caregivers of elderly individuals with disabilities. Low back pain as a chronic pain significantly reduces their physical functioning (e.g., difficulty in daily caregiving actions such as bending and lifting), leading to decreased caregiving efficiency and emotional depletion; on the other hand, family caregivers who have to reduce their caregiving hours or seek alternative care (e.g., hiring temporary caregivers) due to low back pain may further exacerbate the strain on the family's caregiving resources. The underlying reason may be that musculoskeletal injuries, with LBP often serving as an early symptom, are chronic and cumulative in nature. As the quality of life for patients with disabilities declines over time, the complexity of caregiving increases, placing greater physical demands on caregivers. Prolonged adoption of specific postures and repetitive weight‐bearing activities further contribute to chronic musculoskeletal injuries (Yıldırım Kalabalık et al. 2024). Different contexts, populations and caregiving experiences could account for disparate outcomes among studies. Firstly, differences in support systems for family caregivers in different countries may significantly affect the actual burden of low back pain. For example, in Western countries (e.g., Sweden, the United States), ‘community care delivery systems’ are commonly in place, and family caregivers have easy access to skilled nursing equipment (e.g., power lifts), short‐term respite services (respite care in an institution), and occupational health insurance (which covers treatment for LBP), which may reduce the severity of LBP due to long‐term care; whereas family caregivers in the region of this study (or other developing countries) are more likely to rely on their own support systems for LBP. In contrast, family caregivers in the study area were more dependent on their own care and lacked these supports, resulting in a higher risk of low back pain for the same number of years of care. Second, family caregivers often take the initiative to provide long‐term care (e.g., children providing home care for their disabled parents) due to more traditional concepts such as ‘prioritization of family responsibilities’ and ‘filial piety’, resulting in generally longer caregiving years. The very act of long‐term caregiving can increase the load on the lower back, ultimately leading to more severe chronic injuries.

The next significant factor was safe work behaviour. Safe work behaviour included in this study were assessing the patient and risk, assessing and correcting the physical environment (e.g., space or obstacles), using good ergonomics, and obtaining necessary help and patient cooperation from others. In this study, high safety of patient handling behaviours reduced the risk of LBP by 74.6% (OR = 0.254) compared to family caregivers with low safety. Compared to the results of an ICU nurses' survey conducted by Lee et al. (2010), the average score on each SWB‐PH entry for family caregivers of patients with disabilities was (3.64 ± 0.54), considerably less than the ICU nurses' score of (4.94 ± 0.66). First, it may be that the two study subjects are different: hospital ICU nurses as professional caregivers have occupational safety and protection awareness and knowledge base, while in the home environment, most family caregivers are non‐professional and less educated, lacking an understanding of their safety and protection as well as knowledge of low back safety and weight lifting techniques, so the latter caregiving safety behaviours are less safe and more prone to LBP. Second, there may be differences between the two study settings: mechanical patient transfer devices are available in foreign ICU wards, such as freestanding or portable whole‐body mechanical lifts, sit‐to‐stand assistive devices, transfer sheets and wearable lumbar devices (Gold et al. 2017; Iwakiri et al. 2021) whereas the lack of mechanical device‐assisted lifting in the domestic home setting is usually manually lifted by the family caregiver alone, thus leading to low safety of caregiving behaviours of family caregivers for patients with incapacitation, which in turn leads to an increased risk of developing LBP.

This study found that perceived social support was another significant factor for the occurrence of LBP among family caregivers. The results of this study showed that for every 1‐point increase in PSSS, the odds of LBP for family caregivers will be reduced by 5.1%. The previous study also confirmed this in a population of 155 patients with chronic low back pain at Cape Coast Teaching Hospital in the Central Region of Ghana (Ankomah et al. 2023). Several pathways may explain this association. Social support may buffer caregiving related stress and reduce perceived caregiver burden, both of which can influence pain perception and coping within the biopsychosocial model of chronic pain (Cohen and Wills 1985; Gatchel et al. 2007). Support from family members, friends, or other important sources may also provide opportunities for rest and shared caregiving, especially for physically demanding tasks such as repositioning, transferring and lifting patients. This practical support may reduce cumulative lumbar strain during daily care. Evidence from family caregivers of older adults with disabilities in China also suggests that higher social support is related to lower caregiver burden (Zhong et al. 2020). Thus, family caregivers with higher perceived social support may experience less psychological strain and receive more help with patient handling tasks that they would otherwise perform alone, which may partly explain their lower odds of LBP. These mechanisms should be interpreted as plausible explanations rather than causal pathways because of the cross sectional design. Additionally, these findings should be interpreted within China's family based care context. Family members remain the main source of long‐term care for disabled older adults, whereas formal long‐term care and community based services remain unevenly developed (Feng et al. 2020). Filial piety may reinforce adult children's perceived responsibility to provide direct care, while the one‐child policy, smaller nuclear families, urbanization and migration may reduce the number and proximity of available family caregivers (Feng et al. 2020; Zhang et al. 2019). In this broader cultural and structural context, lower perceived social support may reflect fewer opportunities for respite, shared caregiving and assistance with patient handling, which may partly explain its association with higher LBP risk in this study.

### 5.1 Study Limitations and Implications for Future Research

This study has several limitations. First, its cross‐sectional design precludes conclusions regarding temporality or causality. Although associations were identified, the findings should not be interpreted as evidence that repositioning frequency, care years, patient‐handling safety behaviour, or perceived social support causes LBP. The cross‐sectional design also prevented the assessment of changes in caregiving conditions, patient health and caregiver health over time. Second, data were collected using self‐reported questionnaires and may therefore be subject to recall and reporting bias, particularly for LBP during the previous year, caregiving duration, daily care hours and repositioning frequency. Third, convenience sampling of family caregivers from tertiary hospitals in Shenzhen may limit the representativeness and generalizability of the findings, particularly to family caregivers in rural areas, less‐developed regions, or settings with fewer healthcare resources. Consequently, the findings should be generalized with caution and may be most applicable to family caregivers of patients with disabilities in economically developed urban areas of China.

Future longitudinal studies should examine temporal relationships between LBP, care years, repositioning frequency, patient‐handling safety behaviour and perceived social support. Studies should also recruit family caregivers from diverse geographical and socioeconomic settings, including rural and less‐developed regions, to assess the generalizability of the findings and explore regional differences. Furthermore, intervention studies are needed to evaluate whether caregiver training and social support programmes can improve patient‐handling safety behaviour, enhance perceived social support, improve caregiving outcomes and reduce the occurrence of LBP among family caregivers.

## 6 Conclusion

LBP was common among family caregivers of patients with disabilities. Longer care years, more frequent patient repositioning, lower patient‐handling safety behaviour and lower perceived social support were associated with LBP after adjustment for gender, age and regular physical exercise. Given the cross‐sectional design, these findings should not be interpreted as evidence of causality. Healthcare professionals should consider these associated factors when assessing family caregivers and provide appropriate patient‐handling education, practical training and supportive resources. Families and community services should also provide emotional and social support to family caregivers. Further longitudinal and interventional studies are needed to determine whether such measures can reduce the risk of LBP and improve family caregivers' quality of life.

## 7 Relevance for Nursing Practice

Family caregivers should be included in routine nursing assessment during discharge planning and community follow up. Nurses can assess caregiving duration, patient repositioning frequency, patient handling safety behaviour and perceived social support to identify family caregivers who may require additional support. Before discharge, nurses should provide individualized demonstrations and return demonstrations of safe repositioning and transfer techniques. During community follow up or home visits, nurses can reassess caregiving demands, safety practices, LBP symptoms and social support needs. Family caregivers reporting persistent LBP or substantial physical burden should be referred for further clinical assessment and community support. These measures may strengthen caregiver support and reduce potentially harmful physical demands during long‐term family caregiving.

## Author Contributions

**Li Dai:** visualization, validation, conceptualization, project administration. **Jiao Xiong:** supervision, validation. **Yu‐Jiao Sun:** conceptualization, methodology, software, data curation, writing – original draft, writing – review and editing, visualization, project administration, formal analysis, investigation. **Su Liang:** validation.

## Funding

The authors have nothing to report.

## Ethics Statement

The ethical approval for this study was obtained from the Ethics Committee of The People’s Hospital of Baoan Shenzhen and the approval number was (BYL20220508).

## Consent

There was no patient or public contribution to the design, conduct, reporting, or dissemination plans of this study. Family caregivers participated as research participants by completing the study questionnaires.

## Conflicts of Interest

The authors declare no conflicts of interest.

## Supporting information


**Table S1:** Sensitivity analysis using the continuous SWB‐PH mean score in the adjusted binary logistic regression model for low back pain among family caregivers of patients with disabilities (*n* = 209).

**Data S1:** STROBE Statement—Checklist of items that should be included in reports of *cross‐sectional studies*.

## Data Availability

The data that support the findings of this study are available from the corresponding author upon reasonable request.
