# Supplementary material for: Prevalence of Low Back Pain and Associated Factors Among Family Caregivers of Patients With Disabilities in Shenzhen, China: A Cross‐Sectional Study Based on Binary Logistic Regression Modelling
Source: Nurs Open. 2026 Sep 22;13(9):e70787. doi: 10.1002/nop2.70787 (PMC13597071; doi:10.1002/nop2.70787)
Supplement: Supplementary file 1 [file NOP2-13-e70787-s001.docx]

**Supplementary Table S1**. Sensitivity analysis using the continuous SWB-PH mean score in the adjusted binary logistic regression model for low back pain among family caregivers of patients with disabilities (n = 209)

| **Variables** | **B** | **SE** | **Wald χ²** | ***p* value** | **Adjusted OR** | **95% CI** |
| --- | --- | --- | --- | --- | --- | --- |
| Age | -0.033 | 0.031 | 1.140 | 0.286 | 0.967 | [0.910, 1.028] |
| Care years | 0.115 | 0.041 | 7.844 | 0.005 | 1.122 | [1.035, 1.216] |
| Gender | 0.146 | 0.442 | 0.109 | 0.742 | 1.157 | [0.486, 2.751] |
| Regular exercise | -0.973 | 0.736 | 1.747 | 0.186 | 0.378 | [0.089, 1.599] |
| SWB-PH mean score (continuous) | -1.492 | 0.376 | 15.784 | <0.001 | 0.225 | [0.108, 0.469] |
| The number of repositioning |  |  | 17.876 | <0.001 |  |  |
| No more than 3 times per day | Ref | Ref | Ref | Ref | Ref | Ref |
| 4 to 7 times per day | 1.597 | 0.497 | 10.323 | 0.001 | 4.938 | [1.864, 13.082] |
| At least 8 times per day | 1.817 | 0.448 | 16.480 | <0.001 | 6.153 | [2.559, 14.794] |
| Perceived social support | -0.057 | 0.013 | 18.470 | <0.001 | 0.944 | [0.920, 0.969] |

Note: Adjusted OR = adjusted odds ratio; CI = confidence interval; SE = standard error; Ref = reference category; LBP = low back pain; SWB-PH = Safe Work Behavior related to Patient Handling Scale. The sensitivity model replaced the dichotomized SWB-PH variable in the primary adjusted model with the continuous SWB-PH mean score; all other covariates were retained. Model fit: Nagelkerke R² = 0.493; Hosmer-Lemeshow χ²(8) = 10.068, p = 0.260.
