# Supplementary material for: Prevalence of Low Back Pain and Associated Factors Among Family Caregivers of Patients With Disabilities in Shenzhen, China: A Cross‐Sectional Study Based on Binary Logistic Regression Modelling
Source: Nurs Open. 2026 Sep 22;13(9):e70787. doi: 10.1002/nop2.70787 (PMC13597071; doi:10.1002/nop2.70787)
Supplement: Supplementary file 2 [file NOP2-13-e70787-s002.docx]

STROBE Statement—Checklist of items that should be included in reports of ***cross-sectional studies***

|  | Item No | Recommendation | Page No |
| --- | --- | --- | --- |
| **Title and abstract** | 1 | (a) Indicate the study’s design with a commonly used term in the title or the abstract   **An analytical cross-sectional study as stated in the Abstract and Methods.** | Pages 1, 5 |
|  |  | (b) Provide in the abstract an informative and balanced summary of what was done and what was found   **Provided in Abstract.** | Pages 1–2 |
| Introduction | | | |
| Background/rationale | 2 | Explain the scientific background and rationale for the investigation being reported  **Included in the Introduction and background.** | Pages 2–5 |
| Objectives | 3 | State specific objectives, including any prespecified hypotheses  **Stated in purpose.** | Page 5 |
| Methods | | | |
| Study design | 4 | Present key elements of study design early in the paper  **Included in the study design, sample and participant under the Methods section.** | Page 5 |
| Setting | 5 | Describe the setting, locations, and relevant dates, including periods of recruitment, exposure, follow-up, and data collection  **Included in the study design, sample and participants under the Methods section.** | Pages 5–7 |
| Participants | 6 | (a) Give the eligibility criteria, and the sources and methods of selection of participants   **Included in the study design, sample and participants under the Methods section.** | Pages 5–6 |
| Variables | 7 | Clearly define all outcomes, exposures, predictors, potential confounders, and effect modifiers. Give diagnostic criteria, if applicable  **Included in the study design, sample and participants under the Methods section.** | Pages 4, 7–10 |
| Data sources/ measurement | 8* | For each variable of interest, give sources of data and details of methods of assessment (measurement). Describe comparability of assessment methods if there is more than one group  **Included in the Data collection and instruments.** | Pages 7–9 |
| Bias | 9 | Describe any efforts to address potential sources of bias  **Described in study limitations and implication for future research** | Page 19 |
| Study size | 10 | Explain how the study size was arrived at  **Stated in the sample and participants, and result section.** | Pages 6, 10 |
| Quantitative variables | 11 | Explain how quantitative variables were handled in the analyses. If applicable, describe which groupings were chosen and why  **Included in the statistical analysis** | Pages 8–10; Table 3, p. 13 |
| Statistical methods | 12 | (a) Describe all statistical methods, including those used to control for confounding   **Included in the statistical analysis**. | Pages 9–10 |
|  |  | (b) Describe any methods used to examine subgroups and interactions | Not applicable |
|  |  | (c) Explain how missing data were addressed   **Included in the statistical analysis** | Page 9 |
|  |  | (d) If applicable, describe analytical methods taking account of sampling strategy | Not applicable |
|  |  | (*e*) Describe any sensitivity analyses | Pages 8, 10 |
| Results | | | |
| Participants | 13* | (a) Report numbers of individuals at each stage of study—eg numbers potentially eligible, examined for eligibility, confirmed eligible, included in the study, completing follow-up, and analysed   **Included in Results.** | Pages 6, 10 |
|  |  | (b) Give reasons for non-participation at each stage | Not applicable |
|  |  | (c) Consider use of a flow diagram | Not applicable |
| Descriptive data | 14* | (a) Give characteristics of study participants (eg demographic, clinical, social) and information on exposures and potential confounders   **Described in general information of study participants section under the title of results.** | Pages 10–12; Tables 1–2 |
|  |  | (b) Indicate number of participants with missing data for each variable of interest | Page 9 (no missing data) |
| Outcome data | 15* | Report numbers of outcome events or summary measures | Pages 10–11; Table 1 |
| Main results | 16 | (a) Give unadjusted estimates and, if applicable, confounder-adjusted estimates and their precision (eg, 95% confidence interval). Make clear which confounders were adjusted for and why they were included   **Included in the results and Table.** | Pages 9, 12–14; Tables 2, 4 |
|  |  | (b) Report category boundaries when continuous variables were categorized   **Included in the results and Table**. | Pages 8, 12–13; Tables 2–3 |
|  |  | (*c*) If relevant, consider translating estimates of relative risk into absolute risk for a meaningful time period | Not applicable |
| Other analyses | 17 | Report other analyses done—eg analyses of subgroups and interactions, and sensitivity analyses | Page 13; Supplementary Table S1 |
| Discussion | | | |
| Key results | 18 | Summarise key results with reference to study objectives  **Included in the discussion.** | Pages 15–18 |
| Limitations | 19 | Discuss limitations of the study, taking into account sources of potential bias or imprecision. Discuss both direction and magnitude of any potential bias  **Described in study limitations and implication for future research under discussion.** | Pages 19–20 |
| Interpretation | 20 | Give a cautious overall interpretation of results considering objectives, limitations, multiplicity of analyses, results from similar studies, and other relevant evidence  **Described in discussion**. | Pages 15–20 |
| Generalisability | 21 | Discuss the generalisability (external validity) of the study results  **Discussed in study limitations and implication for future research.** | Pages 19–20 |
| Other information | | | |
| Funding | 22 | Give the source of funding and the role of the funders for the present study and, if applicable, for the original study on which the present article is based  **Describe in acknowledgement section of title page.** | Title page |

*Give information separately for exposed and unexposed groups.

**Note:** An Explanation and Elaboration article discusses each checklist item and gives methodological background and published examples of transparent reporting. The STROBE checklist is best used in conjunction with this article (freely available on the Web sites of PLoS Medicine at http://www.plosmedicine.org/, Annals of Internal Medicine at http://www.annals.org/, and Epidemiology at http://www.epidem.com/). Information on the STROBE Initiative is available at www.strobe-statement.org.
